# To Analyze Thrill, Define Extreme Sports

**DOI:** 10.3389/fpsyg.2018.01216

**Published:** 2018-07-24

**Authors:** Ralf C. Buckley

**Affiliations:** International Chair in Ecotourism Research, Griffith University, Gold Coast, QLD, Australia

**Keywords:** adventure, outdoor, recreation, tourism, emotion, evolution, exhilaration

## Abstract

Emotions are a signaling system, evolved by providing selective advantage through enhanced survival and reproduction. The selective advantage conferred by thrill or exhilaration, however, remains unknown. Hypotheses, as yet untested, include overcoming phobias or honing physical skills as juveniles, or exhibiting desirability during mate selection. Extreme sports can provide an ethically and experimentally feasible tool to analyze thrill. To use this tool, extreme sports must first be defined in a non-circular way, independent of participant psychology. Existing concepts, from different disciplines, focus, respectively, on drama, activity types, or consequences of error. Here, I draw upon academic and popular literature, and autoethnographic experience, to distinguish extreme from adventurous levels for a range of different outdoor sports. I conclude that extreme outdoor adventure sports can be defined objectively as those activities, conditions, and levels, where participant survival relies on moment-by-moment skill, and any error is likely to prove fatal. This allows us to examine the motivations, experiences, and transformations of individuals who undertake these activities. In particular, it will allow us to examine the emotional experience of thrill, previously studied principally as an aspect of personality, from new neurophysiological and evolutionary perspectives.

## Introduction

Why does thrill exist? Extreme sports can provide an analytical tool to answer that question. To use that tool, however, we must first define extreme, in an outdoor sporting context. Here, I examine that context, and use it to identify a definition that is theoretically coherent, avoids circularity, and can be implemented in practice. I propose this definition as a basis for future analysis of thrill, using participants in extreme sports as experimental subjects.

Human emotions can be studied from many different perspectives. For example, there is extensive research on: recognition and classification (Johnson-Laird and Oatley, 1989; Oatley et al., 2006); behavioral antecedents and outcomes, differences related to individual personalities, and social roles and functions (Turner and Stets, 2006; Niedenthal and Brauer, 2012); biochemistry, physiology, and neurology (Mujica-Parodi et al., 2014; Orsini et al., 2015; McAllister and Nichols, 2018); and in some cases, selective advantages and evolutionary origins (Tooby and Cosmides, 1990; Nesse and Ellsworth, 2009; Sandseter and Kennair, 2011; LeDoux, 2012; Verma et al., 2016; Pacella et al., 2017).

As with any aspect of human bodies and brains, emotions have evolved through past constraints and pressures, creating comparative selective advantages for individuals with particular traits. We have physical bodies with functions and actions, monitored and controlled by senses, brains, nerves and hormones. But we also have the self-perceived psychological sensations known as emotions, which act as integrated signals of interactions between ourselves as individuals, and our environments, including other people. “Emotions control evolutionarily conserved behavior that is central to survival in a natural environment” (Verma et al., 2016).

What specific selective advantages do we gain from each individual emotion? For some emotions, it is not difficult to propose plausible possibilities. Fear, for example, can be a rapid and powerful signal of immediate threat, requiring a so-called fight or flight response (Do Monte et al., 2016). Anxiety signals a computation that there is an above average probability of an unfavorable outcome. One type of love seems to be a signal of an otherwise undetectable immunochemical aspect of genetically optimal mate selection. Disgust, e.g., at a rotting carcass, may represent an integrated signal, based on smell and sight, of substances likely to cause illness or infection. All of these signals could well provide selective advantages. Emotions, however, are indefinitely nuanced and complex. For some emotions, the evolutionary functions are far from clear. Thrill is one such emotion.

## Context

### Why Does Thrill Exist?

How did thrill, exhilaration, and associated emotional responses evolve? What advantage does an individual human gain from the emotion of thrill, that could improve their chances of producing successful offspring, e.g., through extended survival or advantageous mate selection? Thrill has been studied principally in relation to so-called thrill-seeking or sensation-seeking personalities (Self et al., 2006; Zuckerman, 2007; Woodman et al., 2010; Kruschwitz et al., 2012; Monasterio et al., 2016; Baretta et al., 2017; Zheng et al., 2017); and as a driver of behavioral patterns considered dangerous, abnormal, criminal, or addictive (Lyng, 1990; Elmes and Barry, 1999; Franques et al., 2003; Buckley, 2015a). Thrill, however, is part of the normal panoply of human emotions. It can be experienced by anyone. It is used as a component of education (Berman and Davis-Berman, 2013) and therapy (Gass et al., 2012). It is a commonplace component of commercial tourism products and experiences (Gibson, 1996; Buckley, 2012; Pomfret and Bramwell, 2016; Holm et al., 2017; Lee et al., 2017; Smits, 2018).

As yet, however, there is apparently no accepted model for the evolutionary role or selective advantages conferred by thrill. There seem to be only two published hypotheses to date, neither yet tested. Sandseter and Kennair (2011) suggested that thrill might provide a mechanism for children to overcome phobias. Buckley (2016) suggested that the emotion of thrill might lead juvenile individuals to test and practice their skills, honing them for use later, when they could be needed suddenly for survival. If this is indeed the case, then thrill could play the same role for many animal species, not only humans. There is longstanding evidence for play amongst many species, including mock fights in mammals, and aerobatic maneuvers in birds (Lorenz, 1949/1961); but with little evidence regarding their psychological states. Likewise, if thrill evolved so as to hone skills, then we would anticipate that thrill would lose its attractiveness for adults, whose skills are already developed. We should expect that adults would become increasingly risk-averse once they have invested substantially in resources for survival, competition for a mate, and parental care of offspring. This is indeed broadly the case. Some adult individuals, however, continue to seek lifelong thrills, though some other adults consider such behavior childish or irresponsible. A third possible hypothesis, apparently not put forward previously, is that thrill may lead individuals to display capability in risky activities, demonstrating skill as an aid in mate selection processes. This, however, seems less likely, since thrill does not depend on observers.

To analyze the emotion of thrill, we need individuals who experience it, preferably under experimentally controllable circumstances. Thrill has been reported in a wide variety of circumstances (Avanzi et al., 2008; Buckley, 2016). It is not clear, however, whether this breadth shows that thrill is a widespread and undifferentiated emotion, or alternatively, whether there may be many different kinds or variants of thrill, corresponding to different circumstances. That is, there is as yet no taxonomy of thrills. One of the core or archetypal circumstances or categories of thrill, however, is that experienced by participants in adventurous outdoor sports, including those described as extreme. Therefore, outdoor adventure and extreme sports can provide a laboratory for the study of thrill.

The intensity of thrill experienced by participants in outdoor adventure sports, and its relation to fear, varies considerably between individuals and circumstances (Buckley, 2016). The most intense emotion of thrill is likely to be associated with the more extreme sports. There is a risk of circularity, however, if we use extreme sports to study powerful emotions, but define extreme sports with reference to the strength of emotions experienced by participants. Therefore, to use extreme sports as a tool to analyze thrill, we first need to define extreme sports through external physical criteria unrelated to participant psychology. We can then examine the psychological characteristics and experiences of extreme sports participants without risk of circularity.

### Significance of Defining Criteria

My aim here, therefore, is to consider whether there are recognizable, reliable, and repeatable criteria by which an independent observer can determine whether a specific outdoor adventure activity does or does not qualify as extreme; or alternatively, whether each individual participant sets their own criteria and definitions. That is, I consider whether extremeness can be defined as a characteristic of the setting and activity, rather than the participants’ skills and mindsets.

This is a significant distinction, since the psychology of thrill during high-risk outdoor sports has a number of practical and theoretical implications. First, risk acceptance or aversion differs between individuals, and this applies to a wide range of different risks, including social, emotional, psychological, and financial risks, as well as immediate physical risks. Outdoor adventure provides a tool to analyze the psychology of risk aversion. Can we extend this analysis to extreme adventure? Second, the evaluation of physical risk and reward has been a critical component of human history. It still is, for people who live in areas subject to war or terrorism, high crime rates, difficult terrain, or severe weather; and also for individuals in trades and professions such as the military, fire-fighting, emergency services, construction, tree lopping, electrical maintenance, and more. Outdoor adventure provides insights into human psychology and behavior, both individual and social, in high-risk situations; and these insights are relevant to management and safety in those professions. Can more extreme adventure provide more critical insights?

Third, outdoor adventure generates individual rewards, through immediate thrill or rush (Buckley, 2012) and longer-term self-esteem, personal transformation, and social recognition (Brymer, 2005, unpublished; Brymer and Oades, 2009; Brymer and Schweitzer, 2013, 2017a,b; Holmbom et al., 2017; Buckley, 2018). Can extreme adventure help us analyze how these rewards are generated and evaluated? And fourth, outdoor adventure sports and recreation can be highly addictive (Buckley, 2015a; Heirene et al., 2016; Aiken et al., 2018). Are more extreme sports more highly addictive? To answer any of these questions, we need an unambiguous definition of extreme outdoor adventure sports, that does not depend in a circular way on participant psychology.

Terms such as extreme sports and adventure recreation are recent in origin. Indigenous peoples in regions of severe weather and difficult terrain, and European explorers venturing into those same areas, necessarily demonstrated psychological strength and resilience in the face of physical risk. This applies, for example, to polar regions and high mountain peaks, to untracked deserts and forests, and to the world’s great rivers and oceans. It is only recently in human history, however, that individuals have begun to pursue such activities as a deliberate form of discretionary leisure. This raises the key psychological question in extreme sports (Brymer and Schweitzer, 2017a): why do some individuals voluntarily take part in such activities? What do they gain, that outweighs the very considerable costs and risks?

The psychology of participation in activities that involve excitement and physical risk is a central topic of research on outdoor sports, recreation, and tourism. In commercial adventure tourism, the focus is on providing client satisfaction through thrill or rush, while minimizing risk to the tour provider (Buckley, 2012). In individual outdoor recreation, some highly skilled expert practitioners carry out activities that involve far higher levels of risk, and require far higher levels of skill to survive (Brymer and Schweitzer, 2013, 2017a,b; Arijs et al., 2017; Feletti et al., 2017; Frühauf et al., 2017; Feletti and Brymer, 2018). It is these high-skill, high-risk activities that are commonly referred to, in both the academic and popular literature, either as adventure or extreme. Do those literatures differentiate extreme from adventure, and if so, how?

### Terminologies in Different Disciplines

Extreme sports, in the particular context of outdoor adventure activities, is a rather recent term. The only published use identified by Google^®^ Scholar prior to 1990, is in an analysis of the Hungarian health insurance system, identifying a category of excluded risks (Kereszty, 1989). The term has been used extensively, however, for at least the last quarter century, by at least three different sets of stakeholders. These three groups have used the term extreme sports with somewhat different meanings.

In the popular mass media, the term extreme carries a dramatic connotation, not specifically differentiated from adventure more broadly. This applies for, e.g., screenwriters in mass-media movies (Harlin, 1993); authors of popular autobiographical books (Bane, 1996); and journalists in popular outdoor publications (Burke, 1996; Webster, 2011; Fowler, 2016; Ferry, 2017). In the earliest of these, for example (Harlin, 1993), one of the minor characters, proposing to undertake a parachute BASE jump from a mountain under severe winter weather conditions (and subsequently forced to do so at night under gunfire), says “we like it extreme.” This was a movie in the “action crime thriller” genre, with an improbable plot and equally improbable stunts. The “extreme” parachutists provided a backdrop and a small plot link. The focus was on drama, a typical example of “emotional storytelling” in mass-market movies (Cipresso and Riva, 2016). Drama, not activity, is the defining feature.

In the medical literature, and also in the legal literature of medical insurance, the terms “extreme sports” and “adventure sports” have been used jointly and interchangeably, to refer to a particular group of category of outdoor activities. In some cases these activities are listed explicitly. As noted by Brymer (2005, unpublished), each of these activities can be carried out at various degrees of difficulty, danger, and expertise. The medical literature does not consider that aspect, since its focus is on patients as they present for treatment, and on the types, frequencies, and treatment of injuries (Caine, 2012; Sharma et al., 2015; Laver et al., 2017; Caine and Provance, 2018; Ekegren et al., 2018; Emery, 2018).

Because of the focus on injuries, the meaning of extreme sports in the medical literature is quite broad (Caine, 2012; Sharma et al., 2015; Laver et al., 2017; Caine and Provance, 2018), sometimes including activities such as horse riding and mountain biking (Dodwell et al., 2010) as well as, e.g., parachuting and BASE jumping (Feletti et al., 2017); skiing and snowboarding (Graves et al., 2013; Frühauf et al., 2017); surfing (Pikora et al., 2012; Klick et al., 2016); kiteboarding and related sports (Feletti and Brymer, 2018; Morvan et al., 2018); whitewater rafting and kayaking; and climbing and mountaineering (Soulé et al., 2017a,b). This is logical from a medical perspective, since injury rates from horse riding and mountain biking are indeed high, both in total and *per capita* (Ekegren et al., 2018). Medical statistics, however, may not differentiate between a guided horse riding tour at walking speed, and a steeplechase or rodeo. They do not consider psychological experiences such as thrill, except in relation to addictive behavior.

In the academic literatures of psychology and phenomenology, the term “extreme sports” is a recent addition (Brymer, 2005, unpublished; Willig, 2008; Allman et al., 2009; Sparks, 2016, unpublished) to a longstanding literature on outdoor adventure recreation and adventure tourism (Fenz and Epstein, 1967; Brannigan and McDougall, 1983; Ewert, 1983; Hickman et al., 2016; Rantala et al., 2016; Wang and Wang, 2017). Authors such as Arijs et al. (2017), Brymer and Schweitzer (2017a,b), and Holmbom et al. (2017) have used the term extreme, in preference to adventure, for research on particularly high-risk, high-skill activities, such as proximity wingsuit flying. Brymer (2005, unpublished) referred to “activities where a mismanaged mistake or accident would most likely result in death.”

This literature has focussed principally on: the motivations of participants to undertake such activities; their psychological experiences as they do so; the transformational effects of such experiences on subsequent philosophies and lifestyles; and the analogies and correspondences with spiritual experiences (Brymer and Oades, 2009). Thrill is one aspect of these enquiries, but by no means the only one. Not all of this phenomenological literature uses the term extreme sports (Wiersma, 2014). Some authors prefer terms such as high-risk sports (Frühauf et al., 2017). Similarly, not all extreme-sports literature is phenomenological (Chang, 2017). The principal focus of this body of literature, however, has been on participant experience.

We thus have three rather different emphases on the term extreme, in three different literatures: dramatic, in the popular mass media; activity-specific, in the medical and legal literature; and psychological, in academic analyses. While these three approaches are broadly congruent or at least not directly conflicting, they are by no means identical. In particular, only the third of these, and only a small number of authors in that field, distinguish “extreme” from “adventure.” Given the relationship between fear and thrill (Buckley, 2016), this distinction is an important one, if we are to use outdoor adventure and extreme sports to analyze the origins, mechanisms, and potential selective advantages of thrill as a human emotion. Here, therefore, I set out to determine whether extreme can be distinguished from adventure according to reliable and unambiguous criteria.

## Materials and Methods

I adopt a three-step approach. First, I aim to demonstrate that there is indeed a generally agreed distinction between activities considered to be extreme, and those considered to be adventurous but not extreme. To achieve this, I summarize some examples from both academic and popular published materials, including online and multimedia sources, that can be classified unequivocally into one category or the other. That is, I show that there are some outdoor adventure activities that are typically not considered extreme by any of the participants involved; and in contrast, there are some that have only been attempted by a single or a few persons, and are considered extreme by everyone aware of their existence. A few of these examples, principally in the adventure rather than the extreme category, are also supported by my own autoethnographic experience.

Second, using multiple outdoor adventure activities, I attempt to identify characteristics specific to each of those activities, that would distinguish extreme from adventurous levels. The activities examined are: surfing; skiing and snowboarding; whitewater kayaking; hang-gliding; parachute jumping; and rock and ice climbing. These are congruent with activities considered as extreme sports in the popular, medical, and psychological literatures. Of these, information on surfing, snowboarding, kayaking, and hang-gliding is derived in part from my own autoethnographic experience, as well as the literature and lore of these activities. For parachuting and climbing, I have limited personal experience, and information is derived principally from published sources. I use this information to extract general principles or features of extreme-level practice, that apply across multiple outdoor adventure activities.

Third, I test these criteria by comparing two autoethnographic examples or cases, one of them barely on the extreme side of the adventure-extreme divide, and the other barely on the adventure side. That is, in contrast to the first step outlined above, where I illustrate widely different adventurous and extreme activities in order to demonstrate that this divide exists, in this third step I illustrate activities that are close to the divide, in an attempt to pinpoint what defines it. Finally, I combine these three steps to identify minimal necessary and sufficient defining characteristics of extreme outdoor activities, and consider what this implies for the psychology of high-risk actions, lifestyles, and professions.

Each of these components includes an autoethnographic element, in addition to analyses of published literature and online materials. Autoethnographic approaches can provide particular advantages in the analysis of emotions and associated experiences (Buckley, 2015b, 2016). Autoethnography is now a well-established technique in psychological research (Anderson, 2006; Chang, 2016; Jones et al., 2016; Loftus, 2017; Winkler, 2017). For these components, I complied with: the autoethnographic research protocols put forward by Tolich (2010); the Human Ethics Research requirements and procedures of Griffith University; and the Australian National Code of Conduct for Human Ethics in Research. As an autoethnographic study, conducted in a public space, with no interviews, no inducements, no identification of any observed persons, and no experimental behavior modifications, this research is compliant with all of these protocols without the requirement for specific approvals or consents.

## Results

### Activities With Consensus Classification as Either Adventurous or Extreme

Many adventure activities are available through guided commercial tours or non-profit recreational groups. Most of these are at a low-skill, low-risk level where participants are drawn as much by externally oriented social motivations, as by internally oriented achievement and self-esteem (Buckley, 2012, 2017, 2018; Pomfret and Bramwell, 2016; Rantala et al., 2016). Except in rare cases, the design of these activities provides large safety margins, even for inexpert and inexperienced practitioners. Examples include: guided single-day hikes or walks in easy terrain and comfortable climates; fully equipped and guided raft trips, in warm climates, down short sections of rivers that include only easily swimmable rapids; single-day sea-kayak trips in calm subtropical seas; and so on. While these may be marketed as adventure, and may indeed be perceived as adventurous by urban participants with no relevant prior experience, they would not be classified or marketed as extreme.

At the other end of the scale, there are individual exploits that have been attempted only rarely, or in some cases only once, and which would be regarded as extreme both by expert practitioners and by the general public. Through social media and the greater availability of lightweight digital video recording devices, many of these are now much more visible to the general public than was historically the case. Skiing solo across the Arctic ice toward the North Pole, swimming between moving ice floes in a survival suit (Franco, 2010), is an extreme activity in anyone’s estimation. The same applies to skiing solo across Antarctica (Levy, 2017), or skiing the length of the sub-Antarctic Georgia Island, or skiing down Mount Everest (Nyznik, 1975), or speed-skiing with a kite down the mountain ranges of Alaska (Red Bull, 2014).

Similarly, surfing extremely large, powerful and dangerous waves such as Teahupo’o in Tahiti, Shipstern in Tasmania, Australia, or Nazaré in Portugal (BBC News, 2018), is possible only for the most skilled and experienced surfers, and is recognized as extreme. In whitewater kayaking, there are rivers such as the main gorge of the Yarlung Tsangpo in Tibet (Shangri-La River Expeditions, 2018), or the Inga Falls section of the Congo (Fisher, 2013), that have only ever been attempted once or twice; and others such as the Grand Canyon of the Stikine in Canada that have become legendary tests of skill and courage (Spring, 2012).

These examples, and many more, show that we can indeed recognize cases that are extreme, and cases that are merely adventurous. But these examples alone do not define the dividing line between the two. To take a few examples, is a guided climb to the summit of Mount Everest adventurous or extreme? Difficult and dangerous, certainly; but available as a purchasable product (Boukreev and De Walt, 1997). What about sky-diving onto the North Pole, or steep-slope heli-skiing in Alaska? These are also available commercially, but only to skilled and experienced clients.

What about surfing on large ocean waves such as Jaws (Peahi) in Hawai’i, or Mavericks in California, or Punta Lobos in Chile, or many others? Anyone can paddle out, but they would be foolish indeed unless they already had ample experience in powerful surf elsewhere. Indeed, in the largest surf they will also need a very experienced tow-in jet-ski driver, a specialized tow-in surfboard with footstraps, and extensive practice with both. Similar considerations apply for rivers such as some sections of the Rio Futaleufu or Rio Baker in Chile, or the Mekong, Yangtze and Salween Rivers in China. Anyone can launch a kayak, but without considerable prior experience and expertise, they may not survive. There are commercial tour operations on some of these rivers, but only in less dangerous sections, and only for appropriately qualified clients (Expediciones Chile, 2018; Last Descents River Expeditions, 2018).

So, there is a zone of uncertainty between the adventurous and the extreme. They are not distinguishable automatically. In the next section, therefore, I consider a range of different outdoor recreation activities commonly included in the literature of adventure and extreme sports, and attempt to distinguish features or levels of intensity that would distinguish the extreme from the merely adventurous.

### Patterns Across Activities

Examples of adventurous and extreme options for different outdoor activities are summarized in **Table 1**. For most of these activities, the author is at intermediate adventurous level, nowhere close to extreme. For a few, the author has on occasion (and long ago) ventured slightly into the extreme bracket for some activities, but barely, rarely, and sometimes inadvertently. In whitewater kayaking, the author has never been routinely capable of tackling rivers and rapids considered as extreme, but has, on one or two occasions, paddled rarely run and dangerous rapids that might be considered in that category (Van Beek, 1998). Similarly, the author’s overall kiteboarding skills are intermediate at best, but on a few occasions have included cyclone swells larger than 7 m in size.

**Table 1:** Adventurous cf. extreme, comparison across multiple outdoor activities.

Activity	Adventurous (including tours)	Extreme (individual only)
Surf	Moderate to large sized waves, especially those involving travel to a remote site, but generally in warm water climates, with at least some water depth over seafloor in front of breaking wave.	Very large, shallow, fast, and/or stepped waves, especially over reef or rocky seafloor, and/or hollowing out to drain water from ocean in front of break, and/or in cold water climates. Surfing at night, or in areas with high shark risk, or with frequent floating logs or other obstacles.
Snowboard, ski, heli-ski	Long, steep and/or sloughing powder runs; especially at high altitude; especially with tree wells, rocks or cliffs, but with expert guidance to avoid obstacles; and especially with avalanche risk, but only with avalanche control practices; jumps, but of moderate height, soft and steeply sloping landings, no risk of hitting rocks.	Inescapable couloirs; blind runs down steep and potentially cliffed terrain; runs on sloughing slopes where rider must outrun or outflank sliding snow; runs on avalanche-prone terrain without prior control via bombing; jumps and drops of considerable height, with very precise take-off and landing required to avoid rocks or other obstacles.
Whitewater kayak	Rapids up to Grade IV+ to V, for suitably skilled and experienced paddlers, as long as they are either scoutable or have been run previously, and do not incorporate potentially fatal obstacles, hydraulic features, or large waterfalls.	Rapids at Grade V+ or higher; blind runs through inescapable gorges; large waterfalls; potentially fatal hydraulic features such as inescapable undercuts, strainers, or whirlpools; unavoidable weirs, stoppers, or ledge holes.
Hang gliding, parapenting	Tandem flight in ridge-soaring conditions with steady winds, gentle take-off, and straightforward landings; solo flight by appropriately qualified pilots, including cross-country thermal flights, but only using cumulus cloud streets; hill take-offs from smoothly rounded terrain features, and with bomb-out landing sites in case of need.	Sharp-edge cliff take-offs into strong rising thermals; take-offs from structures such as ramps, bridges and buildings; high-altitude flight; flight in violently powerful thermals; flight during storms; flight using lift from storm fronts, wave clouds, rotating clouds, etc.; inverted and semi-inverted aerobatic maneuvers such as loops and wingovers; cross-country flight over terrain with no landing sites; night flight using landing lights.
Kiteboarding	Winds 10–35 knots (depending on kite size); surf 0–5 m; jumps < 10 m vertical; variety of maneuvers on water or in air, but with bail-out options if they fail.	Winds > 40 knots (depending on kite size); surf > 5 m; jumps > 10 m vertical; high-risk maneuvers such as 360° kite loops; speed-riding (kite plus skis or snowboard on very steep mountain terrain).
Parachuting	Tandem jumps, solo jumps for adequately qualified parachutists; moderate altitude, calm or low wind, daylight hours, safe approaches to landing sites.	BASE jumps, wingsuit proximity flying, high winds, high altitude drops, night jumps, jumps in severe climatic conditions and particularly remote areas (e.g., Arctic); dangerous approaches to landing sites.
Climbing	Rock and ice climbing with adequate skills, equipment and protection, typically grades < 5.10 (depending on climber expertise); sites with safe and straightforward access; weather fine to moderate	Free solos, some bouldering, multi-day multi-pitch big-wall climbs; free-solo vertical or overhanging ice climbs, frozen waterfall climbs; high-graded climbs (depending on climber expertise); remote sites, sites with difficult access and recovery; severe weather conditions.

Examples from hang-gliding include: cliff takeoffs, night flights, one storm front, and aerobatic maneuvers known as wingovers. Cliff takeoffs are risky because they involve running off cliffs blind, at full speed, without knowing what airflow one will encounter. Even with a spotter to watch the movement of vegetation below the cliff as an indicator of airflow, and assistants to steady the hang-glider wing-tips before the run, this is a relatively high-risk move. The author has witnessed one accidental death during a cliff take-off. Night flights were at a ridge-soaring site with a reliable wind and an easy top-landing site, using headlights from two parked cars to mark the cliff edge. This is fine, as long as nothing goes wrong. The storm-front case was not deliberate, but it involved emergency maneuvers to survive a very powerful and turbulent wind. Wingovers are described in the next section.

General features of extreme *cf.* adventurous activities, extracted from the activities in **Table 1**, are listed in **Table 2**. In summary, extreme level activities involve higher skill, focus, and risk. Adventurous activities also involve skill and risk, but if the skill proves inadequate, the consequences are unlikely to prove fatal, unless the participant is unlucky. Extreme activities involve the continuous application of highest-level skills and concentration in order to avoid any error, and any failure is likely to prove fatal, unless the participant is especially lucky. In many activities, any error is likely to cause an immediate and irremediable disaster. Falling on a free solo climb, or hitting a cliff during proximity wingsuit flying, commonly permits no recovery or rescue. This provides a distinction that corresponds to that adopted in previous phenomenological research (Brymer, 2005, unpublished), but is itself independent of the psychology of the participants. We can therefore use that definition to examine the psychology of thrill, without risk of circularity.

**Table 2:** General distinguishing features of extreme cf. adventurous activity levels.

Characteristic	Adventurous level	Extreme level
Available commercially	Yes, can be undertaken either as tour client, or as a private individual	No, can be carried out only highly skilled individuals, independently
Equipment	Own or rented, standard, can be second-hand	Own, best available, often customized
Skill needed to survive	Low (tour) to moderate (solo)	Very advanced, world class
Focus and concentration	Moderate, intermittent	Intense and continuous
Duration of crux moves	Few, short crux points during session	Entire session is continuous succession of crux moves
Consequences of error	Struggle, possible injury	Immediate death likely
Likelihood of death, if error	Unlikely, death only if very unlucky	Likely, would be lucky to survive
Attitude to death	Strongly averse, no expectation	Live to the full, prepared to die

### Illustrations at the Dividing Point

If we define extreme sports through the consequences of any mistake, that raises two further issues. The first is that participants in some voluntary outdoor adventure activities sometimes die not through any mistake of their own, but because of unexpected adverse environmental circumstances that occurred despite accurate prior assessment of low probability. The description by Fiennes (2003) of Scott’s 1911–1912 expedition to the Antarctic provides a carefully analyzed example, although of course that was a scientific exploration rather than a recreational activity. The five men who died on their return from the South Pole were overcome, ultimately, by an unlikely set of weather conditions, that they had no means of predicting or preparing for. Similarly, the death of famed Russian high-altitude mountaineer Anatoly Boukreev in an avalanche was not through any mistake of his own. The factors likely to lead to avalanches are predictable, but the actual occurrence of any avalanche is stochastic. The longer that any individual spends in avalanche-prone terrain, the higher the cumulative risk of being caught by one, irrespective of expertise.

These examples, however, do not conflict with the definition of extreme sports as derived above. There are well-known examples involving multiple deaths, where different individuals, or indeed adventure tourism guides and companies, made different assessments of risks and rewards. These include cases such as deaths during commercial climbs (Boukreev and De Walt, 1997) or canyoning tours (Lashmar and Karacs, 1999).

The second issue is that some individuals may die through accidents even during low-risk adventure activities, even where the activity is routine and oft-repeated, and even where other participants were uninjured. This, however, does not imply that those activities are extreme, but rather that particular individuals were unlucky, or in some cases behaved foolishly. That is, I argue that the distinction drawn here between extreme and adventure sports, based on the likely fatal consequences of any mistake, is still valid overall, even though: (a) some participants in extreme activities die without any mistakes; and (b) some participants in adventure activities die even though the activities are not extreme.

In support of this argument, and to illustrate the precise dividing line between adventure and extreme, I now present one autoethnographic incident that could be judged as (barely) extreme, and one that could be judged as (barely) adventurous. I should emphasize that there are very many activities and participants far more extreme than this example, but that I do not have personal experience of them. I describe these incidents in some detail, in order to convey the sensations experienced from the participant perspective. This is the same approach as that adopted by Buckley (2018), who used one rapid on one river to illustrate the type of incident available to identify broader scale patterns.

The first example is from hang-gliding. It took place many decades ago. A hang-glider is a double-surface aerofoil wing, constructed from fabric over a frame of aluminum alloy tubing braced by wires under tension. The wing exerts substantial drag, and maximum speeds are low compare to cockpit gliders. Hang-gliders have no moving aerofoil surfaces, and they are steered solely by weight shift. The pilot hangs in a harness under the wing, grasping a control bar that forms part of the rigid frame of the hang-glider. If the pilot pushes the bar to left or right, the pilot’s weight causes the wing to bank, initiating a turn. If the pilot pushes the bar forward or pulls it back, the pilot’s weight causes the wing to turn upward or downward, respectively, initiating a stall or dive. Flight angle, speed and direction are monitored by the pilot’s senses, by watching the ground, listening to the sound of air over the wing, and feeling both airflow and gravitational forces on the body.

There are two principal flight strategies: cross-country flight using thermal lift, with radio contact to a pick-up vehicle; or “park’n’play” ridge-soaring flight, using up-drafts along a hill or ridge, at a site allowing take-off and landing at the same location. During ridge soaring, pilots can easily recover altitude, and can therefore practice different maneuvers, such as flying at different speeds, and making tighter or more open turns. For example, a pilot can first gain the maximum altitude available by ridge soaring, and then bank sharply into a series of tight 360° turns, gradually losing altitude in the process, and straightening out into level flight once at the altitude of the ridge, but well in front of it. This maneuver creates substantial G forces and requires concentration, but is relatively safe.

A more risky maneuver is a wingover, a partially inverted aerobatic move. To achieve this, the pilot first gains altitude, and then puts the hang-glider into a steep dive to gain speed and momentum. Once maximum safe speed is gained, the pilot pushes the control bar forward so the wing begins to fly sharply upward, and then banks the wing hard either to left or right, so as to create a steep turn from upward flight. If carried out successfully, this creates a turn where the wing is banked at more than 90° to horizontal. That is, it is a partially inverted turn like a diagonal loop, half-way between a steep turn in normal flight, and a fully inverted loop. A few pilots have indeed completed full loops in hang-gliders, but not using normal flying harnesses.

A wingover involves significant risks. If the pilot does not have sufficient speed on the short section of upward flight, the wing will stall while inverted, causing a crash. Most hang-glider wings can recover from a soft stall during level flight, although there have been fatal accidents when they did not. They cannot, however, recover from a stall when inverted. I did once find myself falling from the sky on top of an inverted hang-glider, and it did not recover, but crashed upside down. Fortunately, on that occasion the distance fallen was small, since I had merely crashed into the top of a tall tree, and fallen out backward. But it does seem that in a hang-glider, stalled inverted flight is unrecoverable.

In a hang-glider wingover, airspeed drops rapidly during the brief period of steep upward flight. The pilot must make an instant judgment, based only on the sound and feeling of airflow, whether or not they have adequate airspeed to support a fully banked turn, allowing for further loss of airspeed during the turn itself. If they do, they can perform a successful wingover. If they do not, they will probably suffer an inverted stall and fatal crash. Once the wingover turn has been initiated, there is no going back.

During an interval of a few moments, therefore, the pilot goes from: (a) diving at high speed, able to see the ground diagonally below and in front; to (b) flying steeply upward, conscious of rapidly decreasing speed but not looking at the ground; (c) a decisive control move, to pull the wing into a sharp bank; (d) the top of the wingover, where airspeed falls almost to zero, the wing falls still and quiet as in a stall, and the ground is visible upside down above one’s head; (e) a near-vertical dive toward the ground at rapidly accelerating speed; and finally (f), pulling out into level flight. The moment of commitment is (c), but the moment determining the outcome is (d), when it is already too late to change your mind.

I tried these a number of times in the mid 1970s. Most were uneventful, though scary. But on one occasion, at the inverted moment (d) as above, my wing actually did stall. Everything went silent, the flying surfaces lost tension, and I heard the sound of the fabric slapping hard against the internal frame. That is an indicator of a hard stall: as may occur, for example, when flying suddenly from calm air into a strongly lifting thermal. The wing dived vertically and began to spin at the same time. Fortunately, it straightened from the spin, and pulled out of the dive the right way up, rather than inverted. So I survived, but only through good fortune. After that incident, I did not attempt any more wingovers.

There are three key considerations in this example. The first is that it was not merely an adventurous activity where something unfortunately went wrong. A wingover is a deliberate move. The second is that the participant knows well that any error is likely to prove fatal, and that they are relying completely on their own skill to avoid any such error. And the third is that once the move has been made, there is no going back. If an error is indeed made, as it was on the occasion described, survival is purely a matter of luck. Therefore, if a line can be drawn between the adventurous and the extreme, we could argue that this event was just on the extreme side.

The second example is from snowboarding. It was during a commercial heli-ski tour, when I was the only snowboarder in the group. In mixed heli groups of skiers and boarders, it is common to let the skiers descend first, since they want to lay down perfect figure-8 tracks on untouched snow. Snowboarders, in contrast, have different ambitions. On this occasion, we were at the top of a large open bowl, running out to a broad shelf, and then dropping in a further slope. To our right was a cornice. Our guide went first, and the skiers followed. And then it was my turn.

I was standing by myself with the whole open slope below me, fresh powder except for a few new ski tracks. My plan was to drop straight downslope, gain speed, and then make a big swooping toe-side turn (i.e., toes pointing downward) to the right, bringing me up under the cornice. There I planned to make a sharp heel-side snap turn back downhill. These are the snowboard equivalents of a bottom turn and cutback when surfing. A large high-speed toe-side bottom turn on powder snow, which may have a radius of 50 m or more, generates powerful G-forces, like a massive weight through one’s knees, and this lets one lay one’s body almost flat to the snow surface. A sudden heel-side snap turn on powder snow throws the full bottom face of the board into the snow, so hard that it carves out a big chunk of snow. These moves are not difficult, but they are fun.

On this occasion, they worked perfectly, and I was heading back downslope, when I suddenly happened to notice that the snow below me had an unusual surface shape, like little waves. I had never seen that before. As I began to wonder how they had formed, I saw that they were getting bigger. At that instant, I realized that I had started an avalanche under the cornice, and the waves were the snow compressing and buckling under the weight of moving snow above and behind me. At the same moment as this realization, I saw the snow surface in front of me divide into a myriad tiny cracks, like a fine network.

I was already riding fast, and I crouched, leaned forward and turned slightly to the left, hoping to outrun the falling snow and escape the main path. As I did so, I saw in front of me, a deep and widening trough in the snow, like the ground yawning during an earthquake. I kicked my back foot down, and jumped the gap at full speed. I was back on firm snow, but from the corner of my right eye I could see house-sized blocks of snow tumbling and accelerating downhill. Ahead I could see a ridge, and I was riding for it at maximum speed. I glanced downslope to check that the skiers were safe, and saw the avalanche halt at the bottom of the bowl, without running across the shelf. They were safe. But from far below me I heard our guide’s voice. “Dooooonnnn’t stooooppppp!” I didn’t.

This was a reputable commercial tour, at an often-used site. We had an experienced guide, and we had taken avalanche safety precautions. It was a large, deep, and violent avalanche, however, powerful enough to have caused severe physical trauma, if I had been caught. In addition, there was nobody upslope to track my path and get quickly to the point where avalanche beacons, probes and snow shovels could have been used. We had a helicopter, but it was shuttling another group, in radio contact but out of sight. Had I been caught, therefore, I should have been lucky to have survived. The risk, however, was derived from an unintended accidental event. The actual snowboarding required was no more difficult than usual. Once again, if a line can be drawn between the adventurous and the extreme, we could argue that this event was on the non-extreme, adventurous side.

## Discussion

Adventurousness, it has been argued (Buckley, 2017), is an individual concept. An outdoor tour may be a lifetime’s most adventurous experience for some of the clients, but a daily routine for the guides. In addition, adventurousness is a subjective concept. An individual who is skilled and fearless at one outdoor activity, may still be a frightened novice at a different activity. How do these considerations apply to outdoor activities at the extreme level? As conceptualized above, extreme activities involve continuous application of highest-level skills and concentration, with any lapse likely to prove fatal. That is, it is the perceptions and actions of the individual participant, moment by moment, that save them from death; and these actions and perceptions are only possible through high-level skill and experience, possessed by few participants. For most participants, though not all, this also involves overcoming fear (Brymer and Schweitzer, 2013, 2017a,b; Buckley, 2016), through remarkable levels of emotional calm, confidence and control.

Is this an objective distinction, independent of individual participants; or a subjective distinction, depending on the attitude of the individual? The chance of making a mistake is influenced strongly by the skill of the individual, as well as the features of the physical environmental setting; but the consequences of any such mistake are influenced only weakly by individual skill, and principally by the physical environment. At the adventurous level, less skilled as well as more skilled participants can commonly survive a fall, wipe-out or corresponding incident. At the extreme level, even the most skilled participants are unlikely to survive. And that is precisely why only the most skilled individuals can participate at extreme levels: there is no room for error, and no second chance. That indicates that, in contrast to the subjective definition of adventurousness, extreme outdoor activities can be defined in objective terms, following the approach first put forward by Brymer (2005, unpublished).

The definition that I propose in using extreme sports to analyze thrill, therefore, reflects past usage in the psychological literature (Brymer, 2005, unpublished) rather than the medical or popular literatures. It relies strongly on objective risk of death in the case of any participant error; or in an equivalent formulation from the reverse perspective, on the critical requirement for continuous exercise of high-level participant skill in order to avoid fatal accidents. It draws a qualitative rather than quantitative distinction between extreme and non-extreme adventure. This contrasts with previous analyses that have conceptualized extreme as one end of a continuum of adventurous activities. The “adventure experience paradigm” (Priest and Bunting, 1993; Jones et al., 2003) argues for a five-step continuum of risk cf. competence that includes “misadventure” and “devastation and disaster.” The formulation adopted here differs from that paradigm. Contrary to that model, I argue that in low-risk adventure, competence is not a critical predictor of outcomes in the event of error. The definition adopted here also contrasts with previous analyses that have used multiple simultaneous criteria, of which risk is only one, to distinguish extreme from adventurous (Lebeau and Sides, 2015). Those approaches include cultural, commercial, and psychological components, contrary to the definition established here.

The definition adopted here contains two key components. The first is that extreme is distinguished from non-extreme adventure by risk rather than activity. In contrast to commercial adventure tourism, extreme outdoor sports involve high physical risk. It is the level of risk, not the type of activity, that distinguishes extreme from run-of-the-mill adventure. The second is that risk is defined as the product of probability and consequences, and the high risk in extreme outdoor sports is high because the consequences of error are severe or fatal. The probability must therefore be low, or the activity could not be carried out at all; and it is low specifically because of the high skill, training, experience, focus and concentration of participants. Commonplace safety measures used during adventure-level activities generally cannot be used during extreme-level activities. They rely purely on participants’ skill, with zero errors.

Both adventurous and extreme outdoor activities may require fitness, training, practice, skill, and split-second perceptions, decisions and actions. At the adventurous level, however, a participant can afford to make mistakes, and still survive. At the extreme level, a single mistake, which can occur in hundredths of a second in some activities, is likely to be fatal. Participants at extreme level cannot afford any mistakes, at all. Therefore, they need the highest levels of skill, capability, control, focus, and judgment. This also sets a psychological distinction between outdoor adventure athletes, and outdoor extreme athletes. The former are adding to the richness of their lives, perhaps accepting a non-negligible risk of injury, but only a very small additional risk of death. The latter believe that life is worth living only if it is lived to the absolute fullest, even at the frequent and significant risk of death. Both experience thrill, but surely, at very different levels. At the adventure level, the thrill is temporary. At the extreme level, it is transformational. This is a distinction deserving of further research.

## Author Contributions

RB designed and conducted the research and wrote the article.

## Conflict of Interest Statement

The author declares that the research was conducted in the absence of any commercial or financial relationships that could be construed as a potential conflict of interest.
